# The Rise and Rise of Medicinal Cannabis, What Now? Medicinal Cannabis Prescribing in Australia 2017–2022

**DOI:** 10.3390/ijerph19169853

**Published:** 2022-08-10

**Authors:** Christine Mary Hallinan, Yvonne Ann Bonomo

**Affiliations:** 1 Department of General Practice, Faculty of Medicine, Dentistry and Health Sciences, Melbourne Medical School, University of Melbourne, Parkville, VIC 3010, Australia; 2 Health and Biomedical Research Information Technology Unit (HaBIC R2), Faculty of Medicine, Dentistry and Health Sciences, University of Melbourne, Level 2, 780 Elizabeth Street, Melbourne, VIC 3010, Australia; 3 Department of Medicine, Faculty of Medicine, Dentistry and Health Sciences, University of Melbourne, Parkville, VIC 3010, Australia; 4 Department of Addiction Medicine, St. Vincent’s Hospital, Fitzroy, VIC 3065, Australia

**Keywords:** medicinal cannabis, pharmacovigilance, monitoring, safety, patient registries, therapeutics

## Abstract

Medicinal cannabis was legalised in Australia in November 2016. By August 2022, there were 5284 specialist physician and general practitioner (GP) prescribers who submitted Special Access Scheme (SAS) applications to the Therapeutic Goods Administration (TGA) for the provision of medicinal cannabis prescriptions their patients. In this article we examine the impact of the delivery of publicly available clinical guidance documents, provision of education to prescribers, establishment of the TGA online portal, and launching of cannabis clinics on the number of applications approved by the TGA over time. We considered these findings in the context of the need to align the interventions facilitating the prescribing of medicinal cannabis with the establishment of processes to enable the systematic monitoring of patient outcomes. The cumulative number of medicinal cannabis Special Access Scheme-B (SAS-B) prescription approvals from January 2017 to June 2022 was 258,926. SAS-B approvals increased at an average rate of 208.55% *p <* 0.000, (95% CI 187.25–229.85) per month. Conclusion: There has been a rapid growth in prescribing since the legalisation of medicinal cannabis in Australia and this expansion has not been accompanied by parallel processes for the monitoring of medicinal cannabis. The capture of more highly granulated data, as found in the electronic medical record (EMR), patient smartphone applications, and social media provide an opportunity to monitor medicinal cannabis effectiveness and safety across multiple prescribing indications.

## 1. Background and Context

Most medicinal cannabis contains one or a combination of two main ingredients from the cannabis sativa plant: cannabidiol (CBD) and tetrahydrocannabinol (THC). THC, a psychoactive cannabinoid, is currently classified as a Schedule 8 (controlled) drug in Australia [1]. Formulations of CBD and THC compounds that contain less than 2% THC and pure CBD are considered non-euphoric and are classified as a Schedule 4 (prescription) medicine [2]. Recent changes to legislation have reclassified some CBD products to a Schedule 3 (pharmacist only) medicine to enable the provision of low dose CBD (<150 mg/day) at a community pharmacy [3]. Scientific evidence for the optimal use of medicinal cannabis is still emerging; hence, medicinal cannabis categorised as an S8 or S4 substance is still classified by Australia’s Therapeutics Goods Administration (TGA) as an unapproved product [1]. 

To date, much of the scientific evidence on medicinal cannabis is obtained from observational data or open label studies rather than from randomised controlled clinical trials (RCTs). Whilst RCTs have long been held as providing the “gold standard” for evidence, pharmacoepidemiology—observational research using routinely collected health data—is increasingly being considered as an approach to gather evidence on the safety and effect of a therapeutic, especially in a rapidly changing environment [4]. To date, the evidence from pharmacoepidemiologic analyses indicate there is a potential therapeutic effect between medicinal cannabis and drug-resistant epilepsy, nausea and vomiting, chronic pain, spasticity associated with multiple sclerosis, insomnia, and palliation [5,6].

The Australian Government legalised the use of medicinal cannabis for the therapeutic management of specific indications on 1 November 2016 [7]. This legislation enabled the production and prescribing of medicinal cannabis for therapeutic and research purposes [7]. Although legalised, most medicinal cannabis products are not listed on the Australian Register of Therapeutic Goods (ARTG); hence, registered health practitioners (prescribers) are required to obtain TGA approval before they prescribe medicinal cannabis products to their patients [6]. Much TGA approval is based on a case-by-case assessment and on the conditions for which medicinal cannabis is prescribed.

Localised pathways for the prescription of, and subsequent access to, medicinal cannabis products in Australian State and Territory jurisdictions were not introduced uniformly. Initially, there was a disjointed process to medicinal cannabis prescribing where the prescriber had to understand not only how to prescribe medicinal cannabis within their local jurisdiction but also how to provide a justification for prescribing. This process was time consuming and convoluted, where the prescribing practitioner had to deliver a paper-based application to both the TGA and their relevant state-based departments for prescription approval. 

In July 2018, a centralised national online portal for medicinal cannabis prescribing was launched [1]. This electronic portal enabled prescribers to lodge online, a single application directly to the TGA for prescription approval. All jurisdictions other than State of Tasmania—where cannabis prescribing was independently managed by the Tasmania’s Controlled Access Scheme—opted into this national online system. Interestingly, whilst Tasmania maintained its state-based approval system for the prescribing of medicinal cannabis, TGA approval data showed much lower rates of medicinal cannabis prescribing per head of population compared to other States and Territories [8,9,10].

Registered medical cannabis prescribers, such as Authorised Prescribers or Special Access Scheme (SAS) prescribers, gain approval via the Authorised Prescriber System or via TGA SAS pathways [6]. Medical practitioners need to apply to the TGA to become Authorised Prescribers; this enables them to prescribe medicinal cannabis directly to their patients via an Authorised Prescriber script. In contrast, SAS prescribers are required to submit a SAS application to the online TGA portal for approval prior to the provision of a prescription to their patient [6]. There are two main SAS prescriber application categories; the SAS-A category provides an expedited pathway for medical practitioners (via the) to prescribe medicinal cannabis to a patient who is classified as seriously ill, and the SAS-B category provides a pathway for registered clinicians (including medical and nursing practitioners) to prescribe to their patient for multiple clinical indications of varied severity [6]. Data included in the SAS-B application include prescriber name, prescriber Australian Health Practitioner Regulation Agency (AHPRA) number, practitioner type, practitioner specialty, consulting location, patient initials, patient date of birth, product type and indication for prescription. Approval for TGA SAS applications can take up to 48 hours or more [6].

Prior to November 2021, Authorised Prescriber and SAS applications for medicinal cannabis products were authorised under a cannabis product trade name; however, since then, medicinal cannabis applications are approved by active ingredient—under one of five categories—based on the proportion of CBD content compared with total CBD and THC content [6]. This approach allows flexibility in brand substitution when required, such as in the event of product shortage or product discontinuation. Previously, prescribers were required to submit a separate application for each different product prescribed [6]. 

By August 2022, of all SAS-A and SAS-B online approvals—278,967—the majority (99.42%) were from SAS-B applications for either single or repeat prescriptions, that had been submitted by SAS-B prescribers. The number of current SAS-B prescribers is 4374, this represents 82.78% of all 5284 SAS prescribers, the other 910 SAS prescribers are recorded on the TGA dashboard as being registered SAS-A prescribers. It is difficult to estimate SAS-B patient numbers because prescription approvals contain provider and product name only; however, patient estimates range from 18,000 to 25,000 [9,11]. The most up to date data on the number of Authorised Prescribers who were currently approved by the TGA to prescribe medicinal cannabis directly to their patients indicates there were 1246 registered Authorised Prescribers in July 2022. 

Of the 277,338 aggregated TGA SAS-B application approvals from January 2017 up to August 2022, most were for chronic pain (59%) and anxiety (23%). There is a much smaller number of approvals for sleep disorders (4%), cancer pain and symptom management (4%), insomnia (3%), neuropathic pain (2%), and post-traumatic stress disorder (2%). Conditions that each represent 1% or less of SAS-B application approvals include that of depression, autism, epilepsy, attention deficit hyperactivity disorder (ADHD), migraine, arthritis, Parkinson’s disease, spasticity, multiple sclerosis, and anorexia [9]. Patient demographic data across the same period showed most patients were aged either between 18 to 24 years (50.00%) or 45 to 64 years (30.88%). Patients aged between 66 to 74 represented almost 9.70% of approvals—this was followed closely by patients aged 74 years or older (7.66%). Patients aged between 2 and 11 years were represented in 0.78% of the data, and those aged from 12 to 17 were represented in 0.92% of the data. There was a negligible number of patients aged less than 2 years (0.06%) [9]. Regarding gender, 57.93% of the SAS-B approvals were for male patients, and 41.52% were for female, all other gender responses were negligible [9]. The highest level of geographic data for prescription approvals was at state and territory level, this was determined by prescriber consulting location [9]. 

**Figure 1:**
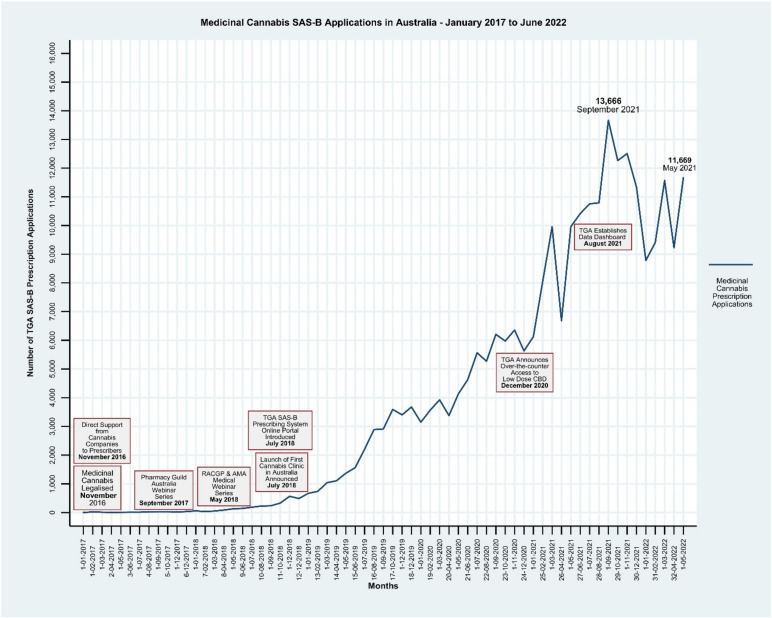
Medicinal cannabis in Australia 2017–2022.

In this commentary, we examine the impact of the delivery of publicly available TGA clinical guidance documents, the provision of education to prescribers via peak professional medical bodies, the development of the TGA online portal, the establishment of cannabis clinics and the launching of the TGA “Medicinal Cannabis Access Data Dashboard” on the number of SAS-B applications approved by the TGA from January 2017 to June 2022 [9]. We consider these findings in the context of the need to align the interventions facilitating the prescribing of medicinal cannabis with the establishment of a process to enable the systematic monitoring of patient outcomes. To do this, we mapped out the milieu and context around SAS-B prescribing in Australia (Figure 1). Firstly, an environmental scan of grey and published literature was undertaken using the search terms “medicinal cannabis” OR “medicine AND cannab*”, “medicinal cannabis” OR “medicine AND cannab*” AND “Australia”. This search provided data sources from the TGA; webinars, presentations, and documents from peak body websites such as the Royal Australia College of General Practice (RACGP), Australian Medical Association (AMA), and Pharmacy Guild of Australia (PGA); medical and pharmacy conference sites; cannabis company websites; news articles; and media releases. 

To augment the “milieu map”, publicly available medicinal cannabis data were accessed from a TGA platform to graphically represent SAS-B approvals [9]. The TGA web-based platform, the “Medicinal Cannabis Data Access Dashboard,” contains de-identified data on the number of unapproved medicinal cannabis products accessed through SAS pathways. For this commentary, the data within the TGA medicinal cannabis dashboard, which are updated monthly, were filtered by date, and downloaded as an Excel file. The data were analysed using Stata 15 software [12]. 

A linear regression was performed on the data to estimate temporal changes in SAS-B prescription approvals over time. The SAS-B data, which included approvals from January 2017 to June 2022, showed a peak number of 13,666 approvals in September 2021 and an average rise in approvals of 208.55%, *p* < 0.000 (95% CI 187.25–229.85) per month. From January 2017 to June 2022 the cumulative number of SAS-B approvals by the TGA was 258,926.

## 2. Considerations

Medicinal cannabis prescribing in Australia has increased substantially over the years since it was first legalised in November 2016. For the first two years following legalisation, there was little prescribing. During this time, specialist and GP prescribers completed and submitted paper-based forms to both the federal regulatory body, the TGA, and relevant State Health Departments, each of which had differing processes for obtaining approval for a medicinal cannabis prescription. In July 2018, a national online portal was introduced for the lodgement of SAS-B applications. This online system streamlined the process for specialist and GP prescribing of medicinal cannabis by enabling the simultaneous submission of prescriptions by prescribers to both Federal and State authorities, namely the TGA and state and territory health departments. All jurisdictions adopted this system for prescribing in 2018, other than the State of Tasmania, which adopted it in July 2021 [8]. At the same time, additional legislation was approved by the Tasmanian Government to permit GPs to prescribe alongside their specialist colleagues [8].

It is evident from the mapping that there was a sharp rise in the rate of prescription approvals in the months following the establishment of the more streamlined online portal system (Figure 1). Other factors that may also have potentially contributed to this rise include continued community discussion and media narrative centered around medicinal cannabis prescribing, the emergence of medicinal cannabis clinics that specialise in the prescribing of cannabis to patients, direct support from cannabis companies to prescribers [13], training courses for GPs and pharmacists on how to prescribe and dispense medicinal cannabis [14], and the establishment of the TGA Data Dashboard [9].

### Monitoring of Access to Medicinal Cannabis in Australia

It is customary to systematically monitor adverse events and long-term effects when a therapeutic is introduced into the community [15]. While data collection on medicinal cannabis approvals is beneficial, there is a need for robust concomitant monitoring on the effectiveness and safety of medicinal cannabis. Ongoing and rigorous monitoring is especially important given that the introduction of medicinal cannabis prescribing into “pharma” was not underpinned by research evidence from clinical trials (as is the usual process for TGA-regulated drugs) [16]. It has been established that the median time to detect adverse event signals from new therapeutics is 4.2 years for pharmaceuticals, and earlier when the introduction is fast-tracked [16]. We can therefore expect, especially considering the rapid rollout of medicinal cannabis, that there are signals that potentially remain undetected. However, evidence from published literature suggests that the adverse effects of medicinal cannabis appear to be manageable and short-lived. These effects are considered to include dizziness, sedation, confusion, and dissociation with THC containing formulations [17] and diarrhoea with formulations of CBD [18]. However, the long-term effects of medicinal cannabis remain unknown and will continue to be poorly understood until signals of longer-term effects are detected. 

In Australia, a patient registry for the monitoring of medicinal cannabis effect and adverse events has not been established. However, the TGA Database of Adverse Events (DAEN), the Australian database for the monitoring of adverse events across all therapeutics [19] shows there have been adverse event notifications relating to medicinal cannabis, albeit these notifications have been minimal. Yet, notwithstanding the value of the DAEN, the monitoring of medicinal cannabis use in the community by a GP could provide another option for the routine pharmacovigilance of medicinal cannabis, especially if monitoring is embedded into a GPs workflow via the GP electronic medical record (EMR) [20,21]. 

Patient Reported Outcomes (PROMs) are also recognised as important facets of the growing knowledge base on medicinal cannabis. Other countries are increasingly engaging consumers in the reporting of medicinal cannabis effects through social media and the use of software applications (apps). Such apps, which provide an opportunity to monitor medicinal cannabis at population level, include those developed in the United Kingdom by Alta Flora to analyse CPASS (Cannabis Patient Advocacy and Support Services) and Project Twenty21 [22,23]. Other novel approaches include the Innovative Medicines Initiative (IMI) WEB-RADR (Recognising Adverse Drug Reactions), a public–private partnership that has been established to develop and evaluate digital tools for the purpose of surveillance and monitoring of therapeutics [24]. The approaches that use and analyse software applications and smartphones are growing in importance and acceptability, especially with improvements in the app-literacy of patients [25,26].

## 3. Conclusions

Since medicinal cannabis was legalised in Australia, there has been a rapid growth in prescribing in the community. However, this expansion has not been accompanied by parallel processes for the monitoring of medicinal cannabis, which makes it difficult to establish its effectiveness in different conditions and difficult to detect side effects or adverse events. The capture of more highly granulated data, such as found in an established registry, would provide the opportunity to monitor product effectiveness and safety across indications and would be especially useful when incorporated with input from prescribers, practitioners, and consumers.

## Data Availability

This study analysed publicly available TGA data https://www.tga.gov.au/medicinal-cannabis-role-tga (accessed on 1 July 2021). Data were analysed using Stata 15 software; coding is available on request.
